# Patients with AML with WT *TP53* but defective TP53-mediated apoptosis have a dismal survival

**DOI:** 10.1172/jci.insight.197261

**Published:** 2026-01-27

**Authors:** Josephine Dubois, Anthony Palmer, Darren King, Mohamed Rizk, Karan Bedi, Kerby A. Shedden, Sami N. Malek

**Affiliations:** 1 Department of Internal Medicine, Division of Hematology and Oncology,; 2 Department of Pediatrics,; 3 Department of Biostatistics, and; 4 Department of Statistics, University of Michigan, Ann Arbor, Michigan, USA.

**Keywords:** Cell biology, Oncology, Apoptosis, Leukemias, p53

## Abstract

The survival of patients with acute myelogenous leukemia (AML) carrying mutations in *TP53* is dismal. We report the results of a detailed characterization of responses to treatment ex vivo with the MDM2 inhibitor MI219, a p53 protein stabilizer, in AML blasts from 165 patients focusing analyses on patients with WT *TP53*. In total 33% of AML were absolute resistant to MDM2 inhibitor–induced apoptosis, of which 45% carried *TP53* mutation and 55% were *TP53* WT. We conducted array-based expression profiling of 10 resistant and ten sensitive AML cases with WT *TP53* status, respectively, at baseline and after 2 hours and 6 hours of MDM2 inhibitor treatment. While sensitive cases showed the induction of classical TP53 response genes, this was absent or attenuated in resistant cases. In addition, the sensitive and resistant AML samples at baseline profoundly differed in the expression of inflammation-related and mitochondrial genes. No patient with *TP53* mutated AML survived. The 4-year survival of AML with defective MDM2 inhibitor–induced TP53-mediated apoptosis despite WT *TP53* was dismal, at 19% when *NPM1* was comutated and 6% when *NPM1* was WT. In summary, we identified prevalent multicausal defects in TP53-mediated apoptosis in AML resulting in extremely poor patient survival.

## Abstract 2

In this paper we quantitatively define the AML patients with WT TP53 but defective TP53-mediated apoptosis and show that this group has a dismal long-term survival.

## Graphical abstract

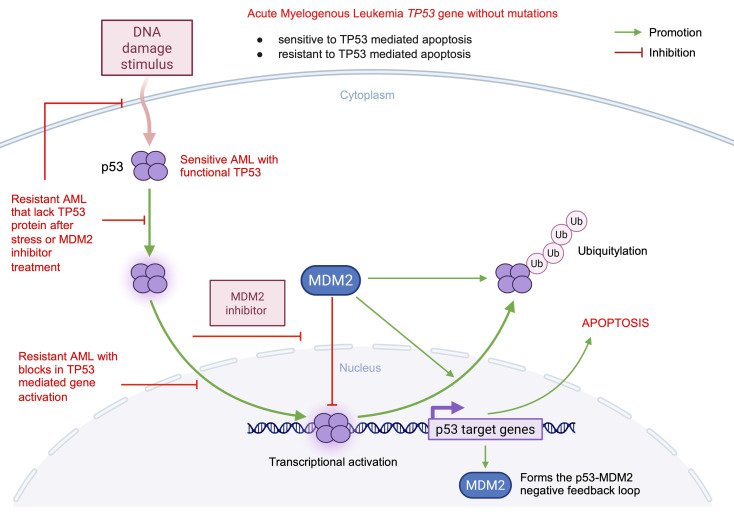

## Introduction

The survival of adult acute myelogenous leukemia (AML) remains unsatisfactory, and novel treatment approaches are needed to improve the outcome of these patients. Of the various high-risk traits that negatively influence AML patient’s survival, mutations in *TP53* stand out as particularly dismal (1–4). Very few patients with AML with *TP53* mutations survive when treated with conventional chemotherapy and the consolidation with stem and marrow cell transplantation also fails to rescue most of these patients.

The 2 major interrelated phenotypes imparted by mutations in *TP53* on AML cells and linked to poor survival are resistance to TP53-mediated apoptosis and highly elevated genomic complexity/instability (3). AML cells carrying *TP53* mutations survive damaging insults, including therapy-induced DNA breaks that would otherwise result in TP53 stabilization, apoptosis induction, and cell death. The damaged cells do not commence apoptosis and, by default, persist with damaged DNA. This damage allows for phenotypic diversification via creation of a clonally diverse genomically unstable AML cell population serving as a substrate for Darwinian selection of more aggressive disease.

While mutations in *TP53* are present in 7%–10% of de novo AML, ~20% of secondary AML and up to 50% of treatment-related AML (tAML) (1, 5), the incidence of nonmutational TP53 defects is less well established and is in part dependent on the assays used for measurements. Equally unclear is whether nonmutational defects in the apoptotic TP53 network are adverse for survival akin to *TP53* mutated AML.

One approach of interrogating the apoptotic TP53 network in cells involves chemical activation via stabilization of TP53 protein through use of drugs that interfere with the binding of TP53 and MDM2 (referred to as MDM2 inhibitors) (6). Multiple compounds that directly interfere with the binding of TP53 and MDM2, including the Nutlins and the MI-series of MDM2 inhibitors, have been developed (6–9). The physical blockage of the TP53 and MDM2 interaction by MDM2 inhibitors results in the elevation of TP53 protein levels, as a prerequisite of TP53-mediated apoptosis or TP53/P21-mediated cell cycle arrest (8, 10–13). Evidence for involvement of intrinsic (e.g., PUMA/BBC3) and extrinsic (e.g. FAS) apoptosis pathways in MDM2 inhibitor–induced apoptosis has been provided, and MDM2 inhibitor–mediated apoptosis seemingly employs redundant apoptotic pathways (14–19).

In this study, we have characterized the apoptotic response of purified AML cells from 59 patients with AML to ex vivo treatment with MDM2 inhibitors and have combined data with previously reported data for 109 patients for a combined total of 165 patients (20). We found that 33% of AML samples were completely resistant to MDM2 inhibitor treatment and that greater than half of these resistant samples carried WT *TP53*. The analysis of gene expression in AML via arrays at baseline and at 2 hours and 6 hours after MDM2 inhibitor treatment demonstrated the impaired induction of TP53 responsive genes in the resistant cases. This phenotype was present irrespective of whether TP53 protein was induced following MDM2 inhibitor treatment or not. The resistant cases predominantly expressed inflammation and cytokine associated gene signatures that likely contributed to the apoptosis blocks. Clinically, the resistance of AML cells to MDM2 inhibitor treatment ex vivo identified a substantial subgroup of patients with AML with 90% mortality.

Overall, we identify a substantial subset of patients with AML with defective TP53-mediated apoptosis and dismal survival. Our data suggest that the prospective identification of high-risk patients with AML with defective TP53-mediated apoptosis is required for improved risk prognostication and for the development of better therapies for this group of patients.

## Results

### Patient characteristics.

Detailed treatment, clinical, survival, and genomic characteristics of the 165 patients with AML with IC_50_ measurements following ex vivo MDM2 inhibitor treatment are summarized in Supplemental Table 1 (supplemental material available online with this article; https://doi.org/10.1172/jci.insight.197261DS1). Of the 165 AML cases analyzed, 145 (88%) were previously untreated and 20 (12%) were relapsed at study enrollment. In total, 73% (120 of 165), 17% (29 of 165), and 10% (16 of 165) were either de novo, secondary, or tAML, respectively.

### Defective TP53-mediated apoptosis is common in adult AML.

The E3 Ubiquitin Protein Ligase MDM2 is the major negative regulator of TP53 protein levels in cells (11–13). We employed the MDM2 inhibitor MI219, which competitively blocks the MDM2 and TP53 protein interaction as a tool to stabilize TP53 protein (20). Next, we measured the TP53-mediated apoptosis induction in purified AML blasts. We purified blasts from 56 AML specimens to > 90% purity and incubated cell aliquots for 40 hours with escalating concentrations of MI219 (20). The apoptotic cell fraction in treated samples was quantitated through FACS analysis based on annexin V–PI and normalized to measurements in paired untreated cells. We combined the data with previously published results for *N* = 109 primary AML for a combined total of *N* = 165 AML (20).

**Figure 1:**
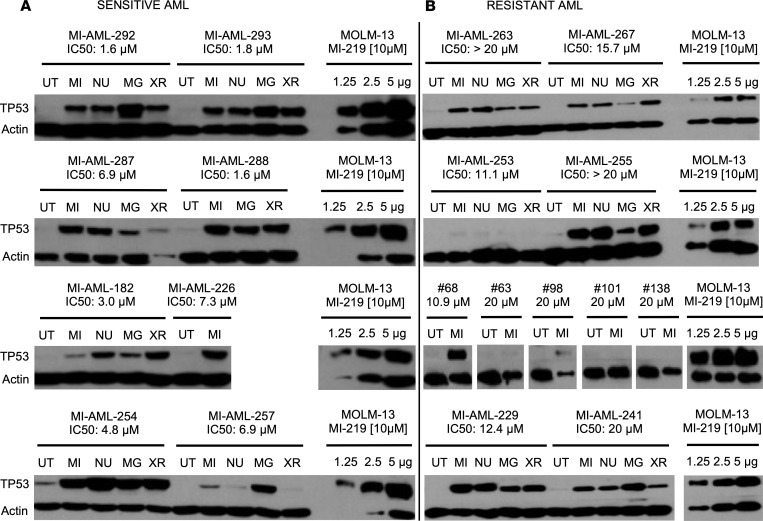
Results of TP53 immunoblotting in sensitive and resistant purified primary human AML blasts all WT for *TP53* before and after MDM2 inhibitor treatment. AML blasts were purified through negative selection and either left untreated (UT) or treated for 8 hours with MI219 (MI; 10 μM), Nutlin 3a (NU; 10 μM), MG132 (MG; 25 μM), or 1-time external irradiation (XR; 5 Gy). After 8 hours, cells were lysed and protein fractionated by SDS-PAGE. Each gel was loaded with an aliquot of a MOLM-13 AML cell line lysate as an internal standard (treated with MI219 at 10 μM and loaded as 1.25, 2.5, and 5 μg of lysate). Protein was transferred to membrane and prepared for immunoblotting with an anti-p53 and anti-actin antibody. Films for both, TP53 and actin were developed together. IC_50_ values for MI219 are indicated. (**A**) Immunoblot results for sensitive AML with IC_50_ < 10 μM. (**B**) Immunoblot results for resistant AML with IC_50_ > 10 μM.

There were 25 of 165 (15%) AML cases carrying *TP53* mutations, all of which displayed absolute resistance to MDM2 inhibitor treatment with IC_50_ values > 10 μM. Furthermore, there were 30 of 165 (18%) AML with WT *TP53* that also demonstrated absolute resistance (IC_50_ values > 10 μM) to MDM2 inhibitor treatment. Given the crucial importance of intact TP53 protein to MDM2 inhibitor sensitivity, we proceeded with analysis of TP53 protein expression levels in primary human AML blasts. We performed immunoblotting for TP53 and ACTIN in lysates from 27 of the cases with WT *TP53* and resistance to MDM2 inhibitor treatment and on 52 sensitive cases at baseline and after MDM2 inhibitor treatment. For some of the AML studied, we also treated with the proteosome inhibitor MG132 or external radiation and measured TP53 expression. Positive control lysates were generated from the AML cell line MOLM-13, treated with MI219 at 10 μM for 8 hours, and aliquots of these lysates were run on every immunoblot. We found that 11 of 27 (41%) of resistant cases showed absent to minimal TP53 protein induction, and representative examples are shown in Figure 1 and Supplemental Figure 1. In contrast, TP53 induction was robust in the remaining 16 of 27 (59%) of resistant cases. In comparison, all 52 (100%) AML cases with sensitivity to MDM2 inhibitors robustly upregulated TP53 protein under these conditions (*P* < 0.00001; Fisher’s exact test; Figure 1 and Supplemental Figure 1).

To gain further insights into the mechanisms of low/absent TP53 expression in resistant AML blasts, we reviewed *TP53* mRNA levels as measured via expression arrays. This analysis disclosed rather uniform *TP53* mRNA levels (TP53 exon expression array probe ID 8012257; processed expression array values are on a log2 scale: resistant 6.85 ± 0.56 mean ± SD; sensitive 7.16 ± 0.34 mean ± SD; *P* = 0.09) across all tested cases. There were no significant differences between resistant AML blasts with low/absent TP53 protein following MDM2 inhibitor treatment and blasts with robust TP53 protein induction. This data point to acquired posttranscriptional mechanisms for low TP53 protein levels.

We reviewed the frequency of selected AML driver gene mutations in the 30 resistant AML cases with WT *TP53*: *ASXL1* 13% (4 of 30), *TET2* 23% (7 of 30), *CEBPA* 0% (0 of 30), *DNMT3A* 37% (11 of 30), *IDH1* 3% (1 of 30), *IDH2* 7% (2 of 30), *RUNX1* 7% (2 of 30), *FLT3* 27% (8 of 30), *KRAS* 7% (2 of 30), *NRAS* 3% (1 of 30), and *MLL-PTD* 12% (3 of 25). The frequency of triple mutated AML (*NPM1, DNMT3A*, and *FLT3-ITD*) was low at 3% (1 of 30). The frequency of *NPM1* mutations was 43% (13 of 30) in the resistant cases and 25% (27 of 110) in the sensitive cases (*P* = 0.078, Fisher’s exact test). These data imply that mutant NPM1 protein interfered with TP53-mediated apoptosis possibly via the known effect of NPM1 mutations on TP53 protein subcellular localization (21). However, there were many *NPM1* mutant cases that were sensitive to MDM2 inhibitors, implying added layers of unknown mechanistic complexity.

Given that mutations in *TP53* have been associated with elevated genomic complexity in many cancers, including AML, we reviewed SNP 6.0 array–based acquired genomic copy number (aCNA) changes in the 25 and 30 cases with *TP53* MUT or WT status and resistance to MDM2 inhibitor treatment, respectively (3). We detected profound differences, in that the mean number of aCNA of AML with *TP53* MUT was 16 (range 0 to 39) with a median of 14, while it was 3 (range 0 to 23) with a median of 0.5 in AML with *TP53* WT status. Using a regression model for genomic lesion count with quantitative IC_50_ and dichotomous *TP53* mutation status as predictors, and holding *TP53* fixed at either WT or MUT, we found that — for every 1 μM increment in IC_50_ values — there were 7% more genomic lesions (*P* < 0.002). The model also indicated that when holding the IC_50_ fixed, patients with *TP53* mutations had on average 5.5-fold more genomic lesions (*P* < 0.0001). In a separate regression analyses, we dichotomized the group by IC_50_ value (≥10 μM or <10 μM) and found that for a fixed *TP53* WT status, a patient with an IC_50_ ≥10 μM had 11% more lesions than a patient with IC_50_ < 10 μM (*P* < 0.002). For a fixed IC_50_, a person with MUT *TP53* had 5.6-fold more lesions than a person with *TP53* WT status (*P* < 0.0001). These models had a logarithmic link and modeled the variance as proportional to the square of the mean, as suggested by Pearson-residual diagnostics.

Therefore, apoptosis resistance alone was insufficient to permit DNA damage and structural genomic aberrations to accumulate, a notable difference from *TP53* MUT cases. These data in conjunction with survival data as shown below implicate apoptosis resistance as the primary contributor to dismal patients’ survival in patients with AML with impaired TP53-mediated apoptosis and WT *TP53*.

### Characterization of gene expression at baseline and changes following MDM2 inhibitor treatment in sensitive and apoptosis-resistant AML blasts.

To characterize the baseline expression and transcriptional response to MDM2 inhibitor treatment of purified AML blasts ex vivo, we selected 10 AML cases with high IC_50_ values (resistant cases) and 10 AML with low IC_50_ values (sensitive cases), all carrying WT *TP53* for gene expression profiling (Supplemental Table 1). The resistant cases were selected based on specimen availability and aiming at balancing TP53 protein status (expressed versus not expressed). We had previously measured TP53 protein levels at baseline and after 8 hours of MI219 treatment in these cases (20). The TP53 protein was strongly induced in all 10 sensitive cases. In contrast, in the resistant cases, 4 did not express TP53, 2 expressed minimal amounts of TP53 and 4 demonstrated robust TP53 induction like the sensitive cases.

We performed Affymetrix GeneChip Human Gene 1.0 ST array–based profiling on RNA/cDNA isolated from purified AML blast at baseline (untreated), and after 2 hours and 6 hours of treatment with 10 μM MI219 (60 array measurements in total; Supplemental Table 2). We excluded probes from further analysis that showed low variance. Next, we performed multiple pairwise comparisons of gene expressions in selected groups.

**Figure 2:**
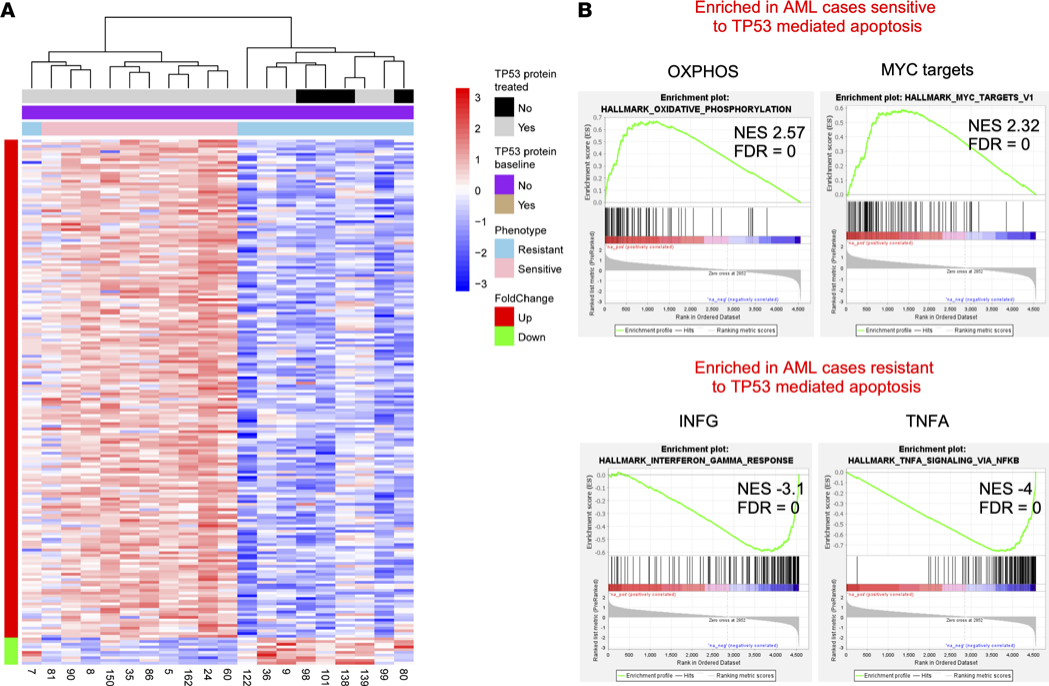
Substantial gene expression differences at baseline in primary human AML with WT *TP53* that are sensitive or resistant to MDM2 inhibitor induced apoptosis. Ten sensitive and 10 resistant primary human AML all WT for *TP53* were purified and incubated with 10 μM of the MDM2 inhibitor MI219 for 0 hours, 2 hours, and 6 hours. Gene expression was measured using GeneChip Human Gene 1.0 ST Arrays (Affymetrix). (**A**) Heatmap: The scaled heatmap displays the genes with > 2-fold changes at FDR < 0.1 between the groups. Results for the unsupervised clustering are indicated at the top. Phenotypes are indicated on the right. The expression of TP53 protein or lack thereof after MDM2 inhibitor treatment is indicated at the top. (**B**) Selected results from Gene Sets Enrichment Analyses (GSEA) (DE genes log_2_-Sensitive/log_2_-Resistant ranked by fold change values) using the hallmark gene sets.

We focused first on the differentially expressed genes between the sensitive and resistant cases at baseline prior to treatment with MDM2 inhibitors (Supplemental Table 3). We display the genes with > 2-fold changes at FDR < 0.1 between the groups in the heatmap in Figure 2A. The unsupervised clustering showed that all 10 sensitive cases clustered together with 1 resistant case, while the other 9 resistant cases formed a separate major cluster. Within the resistant case cluster, the ability to express TP53 protein or not after MDM2 inhibitor treatment did not result in distinct clustering. Using the DAVID algorithm (22, 23), we detected high enrichment scores for mitochondrial genes (ES 11.8, Benjamini-Hochberg [BH] < 1 × 10^–12^) and ribosomal genes (ES 8.4; BH < 1 × 10^–9^) in the sensitive cases. In contrast, resistant cases expressed multiple inflammatory gene signatures (ES 8.2; BH = 1 × 10^–16^) at higher levels. This finding implies contributions by inflammatory mediators and JAK/STAT signaling to TP53-mediated apoptosis resistance in AML (24).

We performed GSEA on the ranked gene list (DE genes log_2_-Sensitive/log_2_-Resistant ranked by fold change values) using the Hallmark gene sets (25, 26). In the sensitive cases, we detected highly significant enrichments for oxidative phosphoryation (OXPHOS), Myc targets, fatty-acid metabolism, and DNA repair (Figure 2B and Supplemental Figure 2), while in the resistant cases, we detected a profound enrichment for inflammatory pathways and mediators, including TNFA, IFNG, IFNA, multiple JAK/STAT signaling pathways, and K-RAS signaling. Furthermore, significantly enriched hallmark gene sets in the resistant cases included “apoptosis” and “p53 pathway” (Figure 2B and Supplemental Figure 2).

The strong enrichment for inflammatory pathways in the resistant cases was notable. The sterile inflammation of AML had previously been described predominantly in monoblastic/monocytic French American British (FAB) M4 and M5 AML and linked with poor outcome (27, 28). In the cases profiled here, 9 of 10 (90%) of the MDM2 inhibitor treatment resistant cases were FAB M4/5, while only 2 of 10 (20%) of the sensitive cases were FAB M4/5. Therefore, our data link defective TP53-mediated apoptosis to sterile inflammation in FAB M4 and M5 AML, thus providing a mechanism of poor survival of monoblastic/monocytic AML.

### Defective expression of core TP53 regulated genes characterizes AML with resistance to TP53-mediated apoptosis.

**Figure 3:**
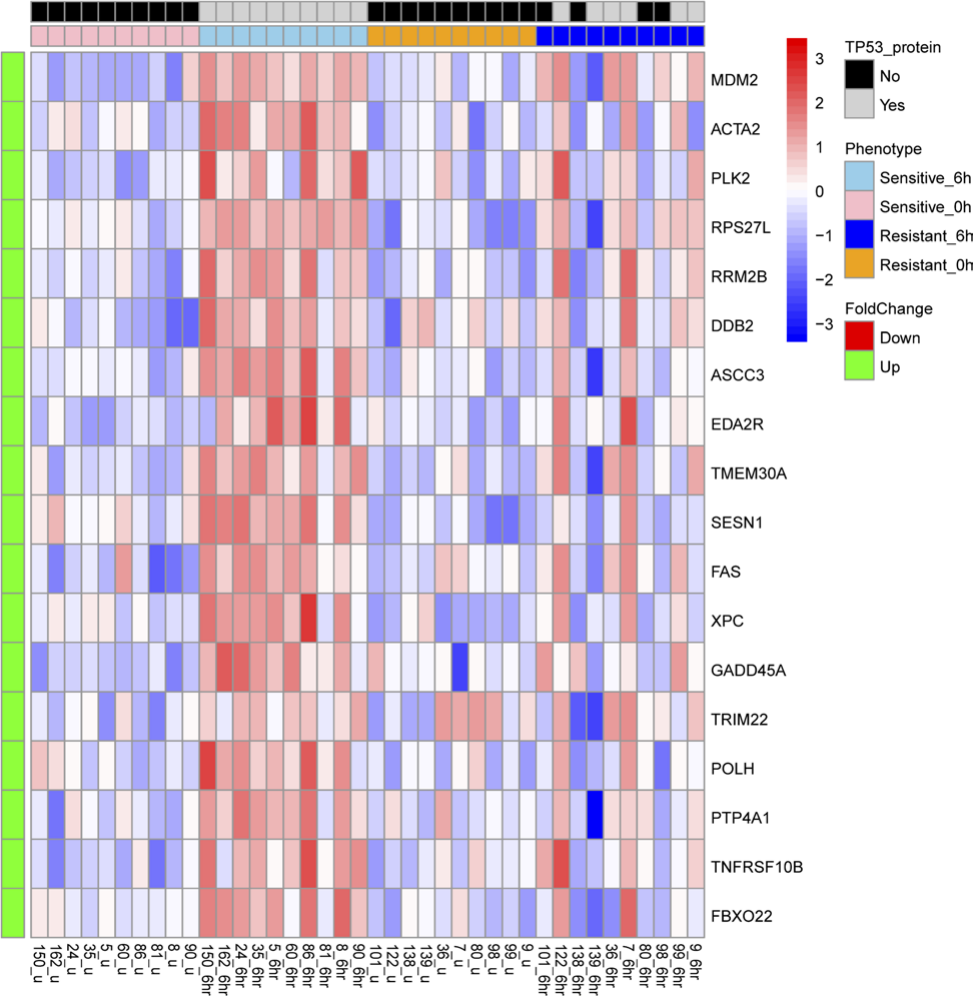
Gene expression changes after 6 hours of MDM2 inhibitor treatment in primary human AML with WT *TP53* that are sensitive or resistant to MDM2 inhibitor induced apoptosis. Ten sensitive and 10 resistant primary human AML were purified and incubated with 10 μM of the MDM2 inhibitor MI219 for 0 hours, 2 hours, and 6 hours. Gene expression was measured using GeneChip Human Gene 1.0 ST Arrays (Affymetrix). In the scaled heatmap, we display the genes with > 1.5-fold changes at FDR < 0.2 at 6 hours in the sensitive cases compared with 0 hours (baseline) and the expression of these genes in the resistant cases at 0 hours and 6 hours, in which none showed significance at FDR < 0.2. Phenotypes are indicated on the right. The expression of TP53 protein or lack thereof before and after MDM2 inhibitor treatment is indicated at the top.

We identified gene expression changes in the sensitive AML cases at the 6-hour MDM2 inhibitor treatment time point (1.5-fold at an FDR < 0.2) (Supplemental Table 4) compared with baseline expression. The list of 18 genes comprised well-known core TP53 transcriptional target genes as compiled from 16 studies by Fischer (29) and displayed in the heatmap in Figure 3. The genes are listed here ordered by magnitude of induction: *MDM2*, *ACTA2*, *PLK2*, *RPS27L*, *RRM2B*, *DDB2*, *ASCC3*, *EDA2R*, *TMEM30A*, *SESN1*, *FAS*, *XPC*, *GADD45A*, *TRIM22*, *POLH*, *PTP4A1*, *TNFRSF10B*, and *FBXO22*. We contrasted the expression of these genes in the resistant cases, in which none showed an induction at the significance threshold of FDR < 0.2 (Figure 3 and Supplemental Table 5). The resistant cases demonstrated gene-dependent and highly variable induction levels and, overall, a much-attenuated induction response than the sensitive cases. One interpretation of these findings is that multiple distinct molecular defects are present in the resistant cases, which affect individual cases and their gene expression differently. Using the same genes, we performed unsupervised clustering and display expression in a heatmap (Supplemental Figure 3). We found that 9 of 10 sensitive cases at 6 hours after treatment clustered together with 2 of the resistant cases at 6 hours after treatment and that the remaining 8 resistant cases at 6 hours after treatment clustered and intermingled with the sensitive and resistant cases prior to MDM2 inhibitor treatment (0 hours, baseline).

We proceeded with 2-way repeated-measures ANOVA to model the effects of drug sensitivity (drug phenotype: sensitive versus resistant) and induction time (0, 2, and 6 hours) within 1 statistical model. We found that almost all of the genes identified above had higher induction slopes in the sensitive cases, and for a subset associated with significant *P* values and FDR < 0.2 (Supplemental Table 6).

**Figure 4:**
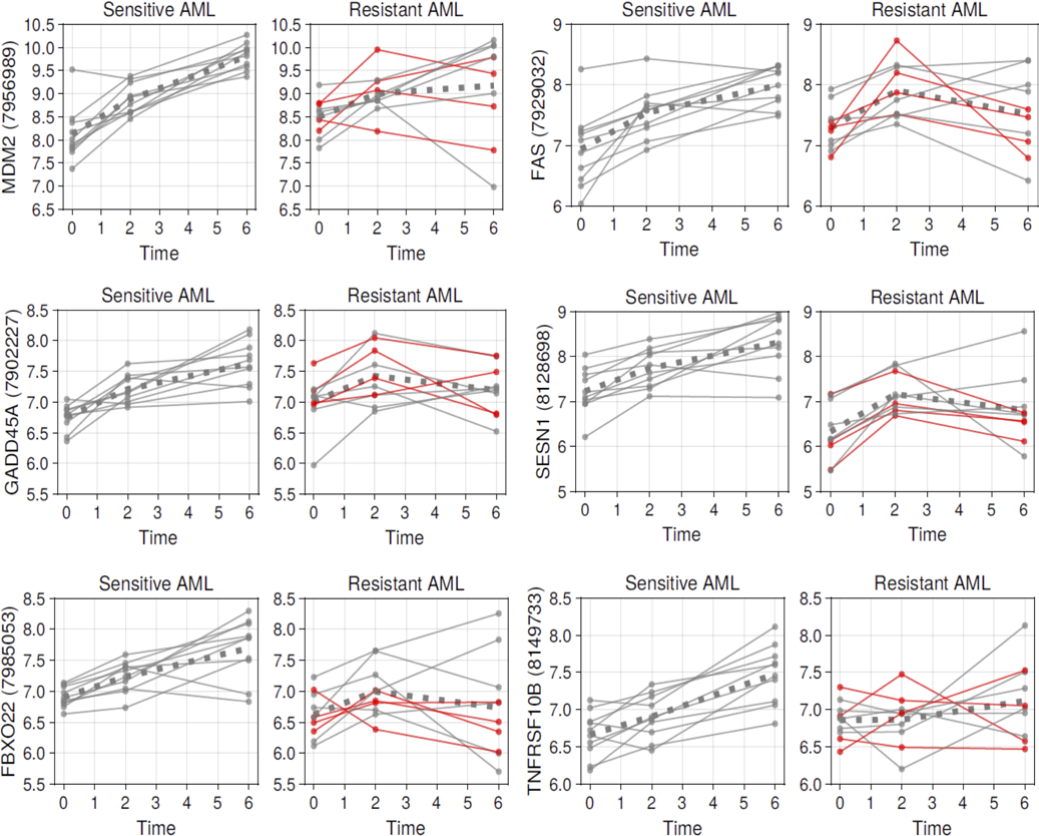
Gene expression induction slopes in 10 sensitive and 10 resistant AML cases of known TP53 inducible genes. Ten sensitive and 10 resistant primary human AML all WT for *TP53* were purified and incubated with 10 μM of the MDM2 inhibitor MI219 for 0 hours, 2 hours, and 6 hours. Gene expression was measured using GeneChip Human Gene 1.0 ST Arrays (Affymetrix). Data for selected genes displayed in (Figure 3) are shown. Red lines: AML that lack TP53 protein expression at baseline and after MDM2 inhibitor treatment. The *x* axis indicates time in hours. The *y* axis indicates array hybridization values and probe ID. Dotted line represents the mean.

To further illustrate these findings, we plotted the gene expression for selected genes at the 0-hour, 2-hour, and 6-hour time points side by side for sensitive and resistant cases (Figure 4 and Supplemental Figure 4). We found that gene expression patterns in the resistant cases were not uniform but instead followed distinct patterns: (a) lack of or attenuated gene induction (e.g., *DDB2*, *MDM2*, *ACTA2*, *RPS27L*, *TRIAP1*, *XPC*, *ASCC3*, *TNFRSF10B*, *RRM2B*, *EDA2R*), (b) gene induction at the 2-hour time point followed by decline (as opposed to further increases as in the sensitive cases) at the 6-hour time point (e.g., *GADD45A*, *FAS*, *IKIP*, *PCNP*, *FBXO22*, *PLK2*, *SESN1*, *PTP4A1*, *TMEM68*), and (c) expression patterns similar to what was measured in sensitive cases (*BAX*, *TMEM30A*). We also compared the gene induction patterns within resistant cases that did or did not express TP53 protein as indicated by the red lines in Figure 4 and Supplemental Figure 4 but found no consistent differences.

Next, we reviewed the expression data for the 2-hour MDM2 inhibitor treatments. Surprisingly, a substantial number of genes were > 2-fold induced at FDR < 0.1 (Supplemental Figure 5, A and B, and Supplemental Tables 7 and 8), and many overlapped between the resistant and sensitive AML groups (Supplemental Figure 5C). We compared these genes with a curated list of 116 high-confidence *TP53* target genes as compiled in Fischer (29*)*. We found only 1 gene (*PLK2*) shared between all 3 gene sets and only 2 genes (*CCNG1*, *RPS27L*) shared between the TP53 gene set and the resistant AML gene set (Supplemental Figure 5D). Therefore, almost all genes with expression changes following 2 hours of MDM2 inhibitor treatment are not high-confidence TP53 target genes and consequently were not further pursued.

### Impaired induction of PUMA (BBC3) in AML with resistance to TP53-mediated apoptosis.

**Figure 5:**
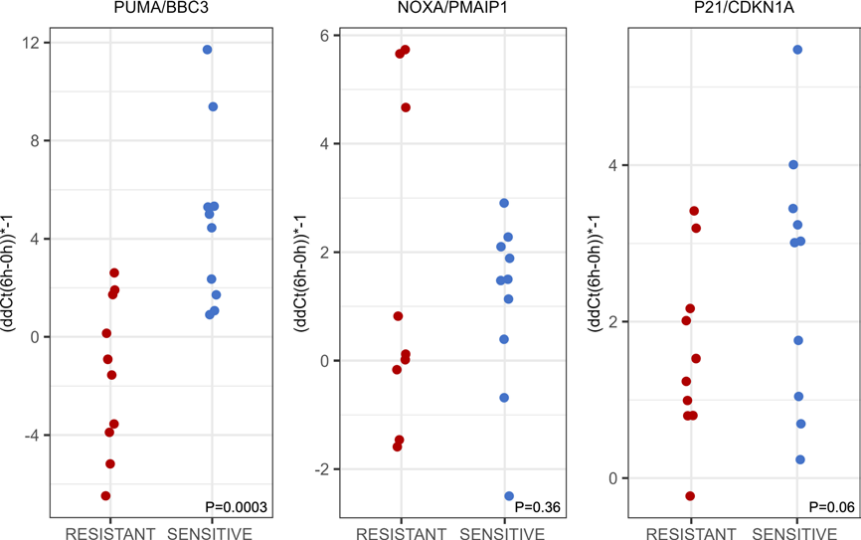
Gene expression of PUMA/BBC3, NOXA/PMAIP1, and p21/CDKN1A in primary human AML with WT *TP53* that are sensitive or resistant to MDM2 inhibitor induced apoptosis. Ten sensitive and 10 resistant primary human AML were purified and incubated with 10 μM of the MDM2 inhibitor MI219 for 0 hours, 2 hours, and 6 hours. Gene expression was measured by qPCR in mRNA/cDNA in technical duplicates. Displayed are ΔΔCT^–1^ based on the 6 hours minus 0 hours ΔCt values. *P* values based on 2-tailed *t* test.

The proapoptotic BH3 proteins PUMA (BBC3) and NOXA (PMAIP1) are TP53 inducible and implicated in TP53-mediated apoptosis (30). Neither of these genes showed substantial changes following MDM2 inhibitor treatment as measured via the gene arrays, which was unexpected. The single *BBC3* probe (ID 8037872) on the exon array targets transcript variant 3, which encodes a BBC3 protein that does not interact with BCL2 thus indicating insufficient *BBC3* transcript coverage. Therefore, we proceeded to measure the normalized expression of both genes via qPCR in RNA/cDNA samples from the samples used for array hybridization. *PUMA* (*BBC3*) was strongly induced at 6 hours in the MDM2 inhibitor treatment–sensitive cases, but the induction was much reduced or absent in the resistant cases (Figure 5). Furthermore, there were only minor differences in the *PUMA*/*BBC3* induction in the resistant cases irrespective of TP53 protein expression, implying both absent TP53 protein and post-TP53 defects in impaired *PUMA* induction, respectively. *NOXA* (*PMAIP1*) overall was not significantly differentially induced between sensitive and resistant cases and therefore unlikely a major apoptosis regulating factor in this setting (Figure 5).

The cell cycle regulatory role of TP53 is mediated by P21/CDKN1A. Using qPCR, we detected induction of *P21* in both the sensitive and resistant cases, albeit with modestly higher amplitude in some of the sensitive cases (Figure 5).

### Varying expression levels of MDM2 and MDMX do not account for the resistance to MDM2 inhibitors in AML.

We had previously measured normalized *MDM2* and *MDMX* mRNA levels across 109 AML as published and detected no correlation with MI219 IC_50_ values (20). Similar findings were obtained when measuring MDMX and MDM2 protein levels in lysates from sensitive or resistant blasts. We reviewed the mRNA expression levels of *MDM2* and *MDMX* in the 20 cases used for the array-based profiling reported here. For *MDMX*, the levels were identical (resistant 8.38 ± 0.49 mean ± SD; sensitive 8.43 ± 0.21 mean ± SD; *P* = 0.4); for *MDM2*, the levels were slightly higher in the resistant cases (resistant 8.57 ± 0.49 mean ± SD; sensitive 8.06 ± 0.39 mean ± SD; *P* = 0.01). Nonetheless, given that the measured resistance to MDM2 inhibition was absolute, the small differences in *MDM2* expression would have been overcome by higher drug concentrations. Therefore, expression levels of MDM2 or MDMX are unlikely the cause for the measured apoptotic defects in the TP53 network in AML.

### A defective apoptotic TP53 network in AML with WT TP53 associates with poor overall survival.

Using the Kaplan-Meier method, we estimated the overall survival for the AML cohort grouped by various known biomarkers. We performed these analyses for the previously untreated group (*N* = 139; UT) and the combined group (*N* = 165; UT + T) in parallel, which gave very similar results. As expected, patients > 60 years of age or carrying high risk genomic karyotypes had very poor outcome. Similarly, patients with ≥ 3 aCNA changes (aCNA = elevated genomic complexity comprised of losses and gains) measured via SNP 6.0 arrays had poor survival (3). There were no long-term survivors (100% mortality) among the patients carrying *RUNX1* mutations in line with previous observations (31) (Supplemental Figure 6, A–D).

**Figure 6:**
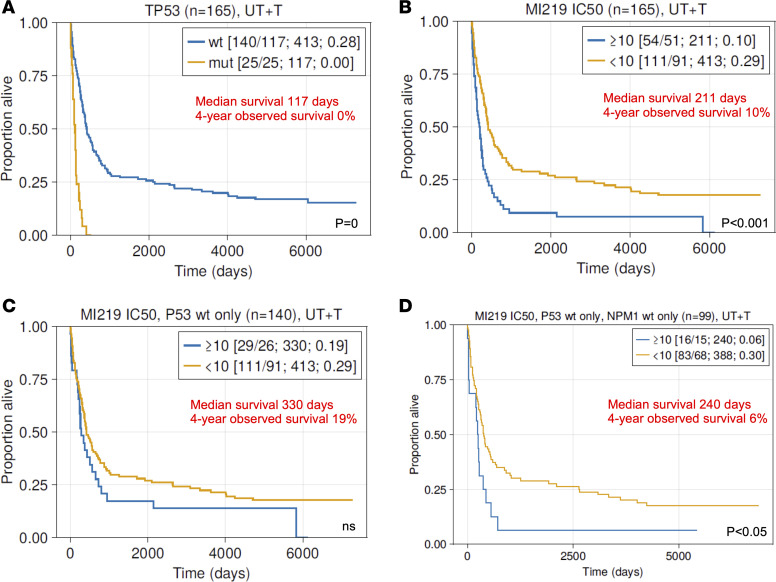
Survival of patients with AML stratified by biomarkers. A cohort of 165 patients with AML with IC_50_ measurements was dichotomized using the indicated biomarkers. (**A** and **B**)*TP53* mutations and IC_50_ value greater 10 μM (resistance) to MDM2 inhibitor (MI219) treatment in AML with MUT or WT *TP53*. (**C**) IC_50_ value greater 10 μM (resistance) to MDM2 inhibitor (MI219) treatment in AML with *TP53* WT only. (**D**) IC_50_ value greater 10 μM (resistance) to MDM2 inhibitor (MI219) treatment in AML with *TP53* WT and *NPM1* WT only. The median survival time in days and the surviving fraction of patients at 4 years is indicated. Survival time distributions were estimated using the Kaplan-Meier method, and comparisons between groups were made using log rank tests.

Next, we reviewed the survival of patients with AML with lack of apoptotic response to MDM2 inhibitors (defective TP53 apoptotic network). There were no long-term survivors (100% mortality) among the patients carrying *TP53* mutations in line with previous observations (3) (Figure 6A). Focusing next on all patients with AML with defective TP53 mediated apoptosis inclusive of all patients with *TP53* MUT as measured by the annexin V–PI flow–based MDM2 inhibitor assays, there were only 3 survivors out of 54 patients (4-year observed survival 10%; Figure 6B). Next, we excluded the *TP53* MUT cases and detected only 3 long-term survivors out of 29 patients (4-year observed survival 19%; Figure 6C). Of note, only 2 of these patients carried *RUNX1* mutations. Finally, once we excluded patients with *NPM1* mutations, the survival of the remaining *TP53* WT patients with AML with apoptotic defects in the response to TP53 activation was 6% at 4 years (Figure 6D).

In summary, patients with AML with *TP53* WT status but impaired TP53-induced apoptosis have poor long-term survival akin to AML carrying *TP53* mutations, thus constituting an understudied high-risk group of patients with AML that is not currently prospectively identified.

## Discussion

This report summarizes detailed studies of adult AML with defective TP53-mediated apoptosis despite harboring WT *TP53*. Through the use of MDM2 inhibitors as tools to stabilize TP53 protein, we have identified 18% of AML out of a large cohort of 165 AML that are resistant to TP53-mediated apoptosis despite WT *TP53*. Through follow-up experimentations, we found the following: (a) AML cases with defective TP53-mediated apoptosis despite WT *TP53* do not fully activate classical TP53 induced genes. This includes the genes *PUMA/BBC3*, *FAS*, *DR5*, and *NOXA/PMAIP1* the principal mediators of TP53 induced apoptosis. (b) The defects or blocks in TP53-mediated apoptosis are multifactorial defying simple categorization schema or reductions to 1 underlying mechanism. These defects include the lack of TP53 expression in 45% of such cases and blocks in the induction of TP53 inducible genes despite preserved TP53 expression in others. (c) AML cases with absolute resistance to TP53-mediated apoptosis despite WT *TP53* when compared with sensitive cases express genes that likely contributed to apoptotic resistance. These included multiple highly enriched gene signatures for inflammatory genes and pathways (TNFA, INFA, IFNG, JAK/STAT signaling) and substantial reductions in mitochondrial genes indicative of reduced mitochondrial mass. (d) AML cases with resistance to TP53 mediated apoptosis despite WT *TP53* have a dismal long-term survival. In aggregate, AML cases with resistance to TP53-mediated apoptosis despite WT *TP53* combined with AML carrying *TP53* mutations comprised 33% of our cohort, emphasizing the unique importance of TP53 abnormalities as resistance factors to conventional chemotherapy and poor prognosis in AML.

As part of these studies, we conducted gene array–based expression measurements in sensitive and resistant primary human AML cases at baseline and at 2 hours and 6 hours following MDM2 inhibitor treatment, resulting in a total of 60 measurements. From these studies, we made the following observations: (a) AML cases with resistance to TP53-mediated apoptosis despite WT *TP53* do not induce classical TP53 response genes or show attenuated induction. A detailed analysis of individual gene induction patterns furthermore uncovered differences between genes providing evidence for multicausal defects in the apoptotic and other TP53 dependent gene expression programs in AML. The latter was true for AML cases that lacked TP53 protein altogether and for AML cases with preserved TP53 protein expression induction. (b) There were substantial gene expression changes at 2 hours in both sensitive and resistant AML cases, the magnitude of which was somewhat surprising. Given that very few of these genes are known TP53 regulated genes they were not studied further. Future work may focus on these gene sets and their relevance to TP53 function and/or TP53-independent MDM2 dependent gene expression. (c) Resistant AML were highly inflamed and of monoblastic/monocytic phenotypes, thus linking a prognostically adverse phenotype to acquired blocks in TP53-mediated apoptosis (24, 28). Ongoing work on the effects of individual inflammatory mediators on apoptosis thresholds will uncover mechanisms underlying this observation.

Focusing on the resistant patients with AML with WT *TP53*, all of which expressed TP53 mRNA at levels similar to sensitive cases, 2 subsets emerged that displayed either low or absent TP53 proteins or preserved TP53 protein levels. Given the scarcity of the primary source material, this was not studied further but raises questions regarding negative regulators of TP53 protein levels that should be evaluated in future studies. Regarding the AML blasts with robust induction of TP53 protein after MDM2 inhibitor treatment, the 2 principal apoptotic molecular defects are a defective TP53 protein, possibly due to aberrant post-translational modifications (32, 33) or defects in the TP53-regulated apoptotic network, which we have measured and confirmed via array based and qPCR based measurements.

Regarding the direct clinical implications of these findings we offer the following conclusions: (a) defective TP53-mediated apoptosis is common in AML and not routinely measured, (b) the defective TP53-mediated apoptosis associated with dismal survival motivating measurements in the clinical setting, and (c) current measurements of biochemical or molecular features in resistant AML are not comprehensive enough to detect all such cases as the underlying molecular defects are diverse and not fully identified (34, 35). Based on the above, one may therefore propose that ex vivo testing of purified AML blasts from patients may offer clinical benefits. Kytola et al. recently demonstrated the clinical predictive utility of ex vivo venetoclax-based apoptosis measurements using flow in AML, and simple modifications of this assay using commercially available MDM2 inhibitors would identify AML with defective TP53 mediated apoptosis (36).

In summary, these data based on the analysis of 165 primary human AML cases quantitatively describe primary resistance to TP53-mediated apoptosis in AML and provides evidence for multiple distinct molecular mechanisms causing this phenotype. The highly adverse effects on AML survival necessitates incorporation into routine clinical care and design of relevant clinical trials aimed at overcoming these defects.

## Methods

### Sex as a biological variable.

Cryopreserved samples from consecutively enrolled male and female patients were analyzed provided sufficient material was available for the various analyses.

### Cell purification.

Mononuclear cells from blood or marrow from patients with AML were isolated by Ficoll gradient centrifugation (GE Healthcare), aliquoted into FCS with 10% DMSO, and cryopreserved in liquid nitrogen. For purification of AML blasts using negative selection, cryopreserved PBMCs were washed and recovered by centrifugation at 500 × *g* and then treated with anti-human CD3 (Miltenyi Biotec #130-050-101), anti-human CD14 microbeads (if blasts were negative for CD14 expression; Miltenyi Biotec #130-050-201), anti-human CD19 (if blasts were negative for CD19 expression; Miltenyi Biotec #130-050-301), and anti-human CD235a (Miltenyi Biotec #130-050-501) per manufacturer’s recommendations. Cell suspensions were run through Miltenyi MACS separation LS columns (Miltenyi Biotec #130-042-401) to negatively enrich for AML blasts. All blast preps were analyzed by cytospins for purity. This schema resulted in greater than 90%–95% blast purity.

### AML blasts MDM2 inhibitor apoptosis assays.

Blasts enriched to > 90% purity were incubated in serum-supplemented RPMI medium at 2.5 × 10^5^ cells in 100 μL final volume in the presence of 0.625–20 μM of the MDM2 inhibitor MI-219 for 40 hours (6). Apoptosis and necrosis were measured for each treated blast aliquot using annexin-V/PI FACS-based readouts and values subsequently normalized to spontaneous death rates in untreated parallel cultures according to the formula (% alive = % mean alive treated samples/% mean alive paired nontreated samples).

### Preparation of purified AML blasts-derived RNA/cDNA for hybridization to affymetrix GeneChip human gene 1.0 ST arrays and data analysis.

Cryopreserved AML derived PBMC samples were thawed and blasts purified using Miltenyi column based negative selection as described and following MDM2 inhibitor treatment (0 hours, 2 hours, or 6 hours) RNA extracted using the Trizol reagent (37). RNA was further purified using the RNeasy kit (Qiagen 74104) and the integrity analyzed using the bioanalyzer platform (Agilent). Total RNA was amplified using the GeneChip WT (whole transcripts) Pico kit (Thermo Fisher #902622) and cDNA hybridized to the GeneChip Human Gene 1.0 ST Arrays (Affymetrix) following the manufacturer’s recommended protocols.

Affymetrix GeneChip data were analyzed as previously described (38). Raw probe-level data were converted to expression measures using the Robust Multi-array Average (RMA) method, which is implemented in the Affymetrix package of Bioconductor (39). Briefly, the raw perfect match (PM) probes were first quartile normalized to reduce array-to-array variation. The normalized probe data were then converted to an expression measure (log_2_ scale) for each gene on each chip. For differential expression analysis relating to MDM2 inhibitor IC_50_ values, we used 2 sample *z* tests to compare the mean log-scale expression level between MDM2 inhibitor sensitive and resistant AML samples. The FDR (40) was calculated, then fold-changes, FDR values, and *z* scores were used to identify genes having a strong association with MDM2 inhibitor IC_50_ values grouped into sensitive and resistant cases. The gene expression matrix for all cases and probes is in Supplemental Table 2.

### Measurement of PUMA and NOXA mRNA expression using qPCR.

RNA was prepared from 10 resistant and 10 sensitive AML cases and converted to complementary DNA using Superscript III first strand synthesis kit (Invitrogen) and random priming. Primers and TaqMan-based probes were purchased from Applied Bio-systems (Primers-on-demand). Primer/probe mixtures included: PUMA (Hs00248075_m1), NOXA (Hs00560402_m1), and human GAPDH.

Duplicate amplification reactions included primers/probes, TaqMan 2x Universal PCR Master Mix, No AmpErase UNG, and 1 μL of cDNA in a 20 μL reaction volume. Reactions were done on an ABI 7900HT machine. Normalization of relative copy number estimates for the mRNA of the gene of interest was done with the Ct values for GAPDH as reference (Ct mean gene of interest – Ct mean GAPDH). Comparisons between time points were performed though subtractions of means of normalized Ct values.

### AML blast treatment and immunoblotting procedures.

Primary human AML-derived blasts were purified as outlined above and subsequently cultured for 8 hours with escalating concentrations (0–20 μM) of MI-219. Cells were harvested and lysed in detergent lysis buffer (50 mM Tris pH 7.5, 100 mM NaCl, 2 mM EDTA, 2 mM EGTA, 1% Triton X-100, 20 mM NaF, 1 mM Sodium orthovanadate [#13721-39-6 Alfa Aesar], 1 mM Phenylmethanesulphonylfluoride [Pierce], phosphatase inhibitor cocktail I [P2850, Sigma-Aldrich] and protease inhibitor cocktail [P8340, Sigma-Aldrich]), protein fractionated, and prepared for immunoblotting with antibodies directed against TP53 (Ab-6, clone DO-1, Calbiochem) and β-actin (AC-15, Sigma-Aldrich). Positive control lysates were generated from the AML cell line MOLM-13 (DSMZ) treated with MI-219 at 10 μM for 8 hours, and aliquots of these lysates were run on every immunoblot.

### Exon resequencing of TP53 and AML driver genes.

Primers to amplify and sequence exon 12 of human *NPM1*, exons 13–15 and 20 of human *FLT3*, exons 2–10 of human *TP53* and adjacent intronic sequences, *ASXL1*, *TET2*, *DNMT3A*, *RUNX1*, *CEBPA*, and known hotspots for *IDH1*, *IDH2*, *K-RAS*, and *N-RAS* were designed using the primer 3 program (http://frodo.wi.mit.edu/cgi-bin/primer3/primer3_ www.cgi). PCR products were generated using Repli-g–amplified (Qiagen) DNA from highly pure blast cells as templates. PCR amplicons were prepared for direct sequencing with internal nested sequencing primers using the exonuclease/shrimp alkaline phosphatase method (USB). Mutation Surveyor (SoftGenetics LLC) software was used to compare experimental sequences against Refseq GenBank or genomic sequences as well as by visual inspection of sequence tracings. Mutations were confirmed using paired patient buccal DNA as templates.

### Statistics.

Linear regression models were used to express the expected log expression of each gene in terms of main effects for linear time (0, 2, 6 hours), sensitivity status (sensitive versus resistant), and their interaction. These linear models were fit using generalized estimating equations (GEE) clustered on person, with independent working correlation structure to account for the longitudinal nature of the data. The interaction term in each model captures the difference of time slopes for the sensitive minus the resistant individuals. The *z* scores (estimated interaction coefficient divided by its standard error) for the collection of all analyzed genes were interpreted using local FDR.

Survival time distributions were estimated using the Kaplan-Meier method, and comparisons between groups were made using log rank tests. Additional information is provided in the legends.

Lesion counts were regressed on MI219 IC_50_ and TP53 status as predictors using generalized linear models (GLM), with a log link function and power-law variance model. The variance model was tuned to the data based on the empirical variance of Pearson residuals within 3 strata of equal size determined by the fitted mean. Additive models and models with a MI219 × TP53 interaction were fit and compared using Wald tests of the interaction term. Separately, dichotomous MI219 was defined based on IC_50_ ≥ 10 versus IC_50_ < 10, and this dichotomous covariate was used in the GLM in place of the quantitative MI219 IC_50_ value. Finally, the data were stratified into 4 groups based on the combined values of dichotomous MI219 and TP53, and lesion counts were compared between each pair of groups using Mann-Whitney *U* tests. Comparisons of ΔΔCT Q-PCR results were performed using 2-tailed *t*-testing.

### Study approval.

The cryopreserved peripheral blood mononuclear cell samples used for this study were from 165 patients with AML enrolled at the University of Michigan Comprehensive Cancer Center between March 2005 and October 2009. The University of Michigan IRB (IRBMED #2004-1022) approved the study, and written informed consent was obtained from all patients prior to enrollment. All investigations were performed in accordance with ethical guidelines outlined in the declaration of Helsinki. The survival or death of patients was ascertained in February of 2025. Detailed characteristics of the 165 patients with AML with IC_50_ measurements following ex vivo MDM2 inhibitor treatment are summarized in Supplemental Table 1. Samples from consecutively enrolled male and female patients were analyzed provided sufficient material was available for the various analyses.

### Data availability.

The CEL files and corresponding biomarker information for the *N* = 20 AML cases (*N* = 60 array files) have been deposited under accession no. GSE297461. A Supporting Data Values file is provided.

## Author contributions

JD, AP, MR, DK, and SNM performed the laboratory research. The clinical data were curated as previously published (3). KB and KAS assisted with statistical analysis. SNM conceived the study and supervised the work. All authors contributed to the writing of the paper.

## Funding support

This work is the result of NIH funding, in whole or in part, and is subject to the NIH Public Access Policy. Through acceptance of this federal funding, the NIH has been given a right to make the work publicly available in PubMed Central.

- NIH R01CA217954
- Clinical Scholars Program of the Leukemia and Lymphoma Society of America (SM)
- NIH through the University of Michigan’s Cancer Center Support Grant (5 P30 CA046592)
- Use of the following Cancer Center Shared Resources: Cancer Data Science

## Supplementary Material

Supplemental data

Unedited blot and gel images

Supplemental table 1

Supplemental table 2

Supplemental table 3

Supplemental table 4

Supplemental table 5

Supplemental table 6

Supplemental table 7

Supplemental table 8

Supporting data values

## Version 1 01/27/2026

In-Press Preview

## Version 2 03/09/2026

Electronic publication
