# Supplementary material for: Patients with AML with WT TP53 but defective TP53-mediated apoptosis have a dismal survival
Source: JCI Insight. 2026 Jan 27;11(5):e197261. doi: 10.1172/jci.insight.197261 (PMC13041678; doi:10.1172/jci.insight.197261)
Supplement: Supplemental data [file jciinsight-11-197261-s023.pdf]

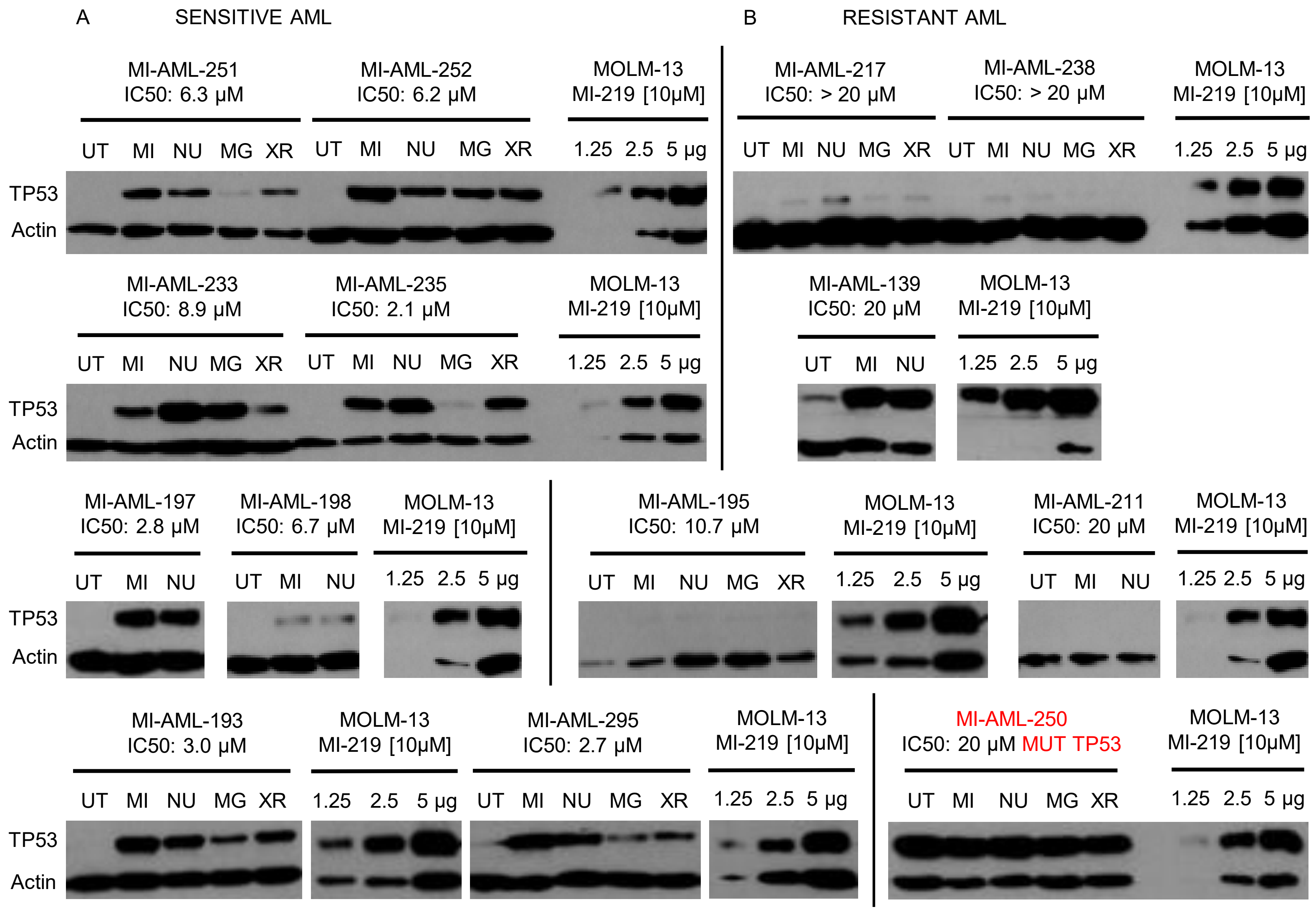

Supplementary Figure 1: Results of TP53 immunoblotting in sensitive and resistant purified primary human AML blasts all WT for *TP53* before and after MDM2 inhibitor treatment: AML blasts were purified through negative selection and either left untreated (UT) or treated for 8 hours with MI219 (MI; 10µM), Nutlin 3a (NU; 10µM), MG132 (MG; 25µM) or one-time external irradiation (XR; 5Gy). After 8 hours, cells were lysed and protein fractionated by SDS-PAGE. Each gel was also loaded with an aliquot of a MOLM13 AML cell line lysate as an internal standard (treated with MI219 at 10µM and loaded as 1.25, 2.5 and 5µg of lysate). Protein was transferred to membrane and prepared for immunoblotting with an anti-p53 and anti-actin antibody. Films for both, TP53 and actin were developed together. IC50 values for MI219 are indicated in brackets. **(A)** immunoblot results for sensitive AML with IC50 < 10 µM, **(B)** immunoblot results for resistant AML with IC50 > 10 µM. Please also see Figure 1. One *TP53* mutated AML case (MI-AML-250) with typical mutant TP53 protein expression is shown.

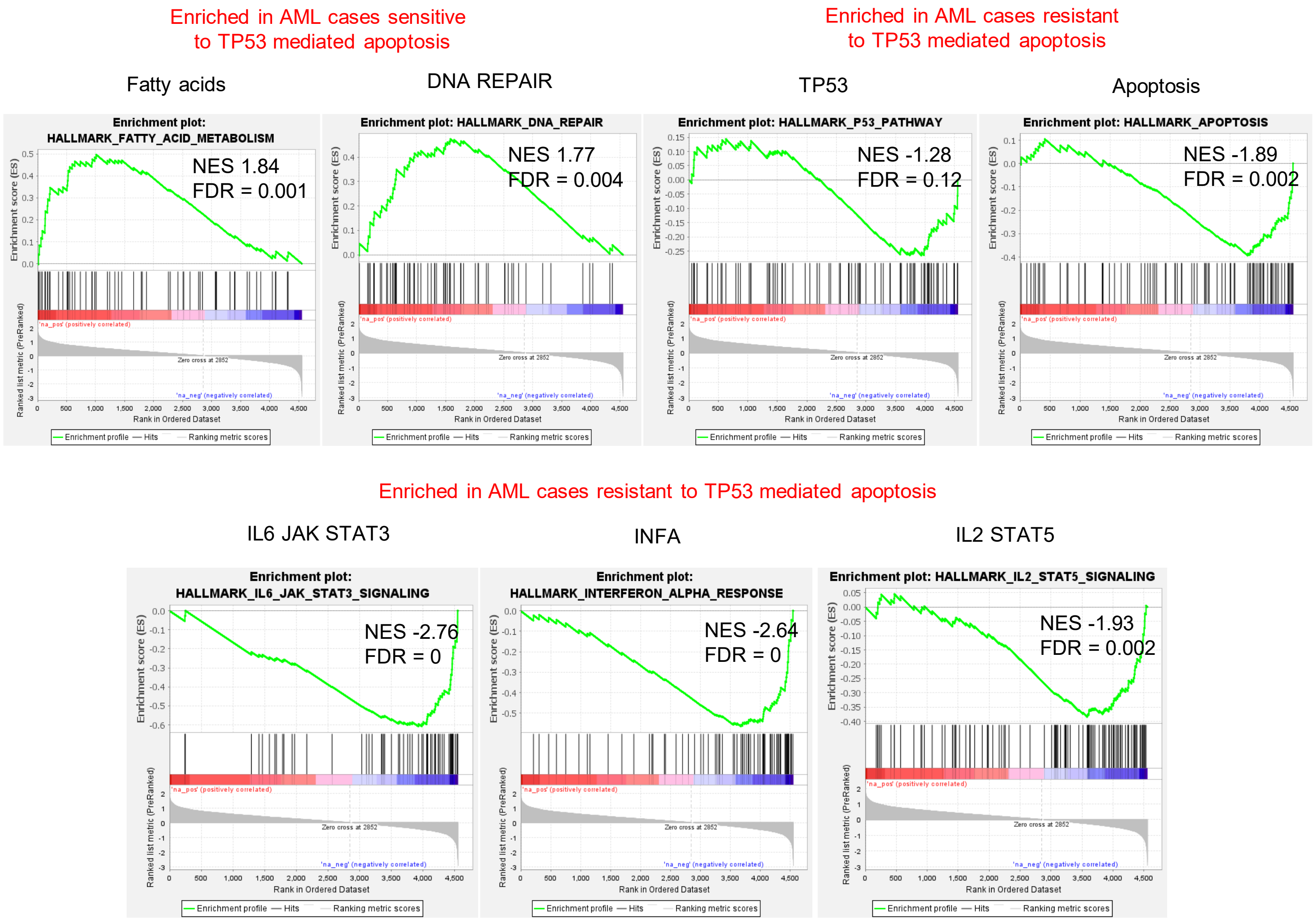

Supplementary Figure 2: Substantial gene expression differences at baseline in primary human AML with WT *TP53* that are sensitive or resistant to MDM2 inhibitor induced apoptosis: Ten sensitive and 10 resistant primary human AML all WT for *TP53* were purified and incubated with 10µM of the MDM2 inhibitor MI219 for 0h, 2h and 6h. Gene expression was measured using GeneChip^{TM} Human Gene 1.0 ST Arrays (Affymetrix). Selected results from Gene Sets Enrichment Analyses (GSEA) using the hallmark gene sets based on fold-change ranked differentially expressed genes between sensitive and resistant cases. NES: net enrichment score, FDR: false discovery rate. Please also see Figure 2.

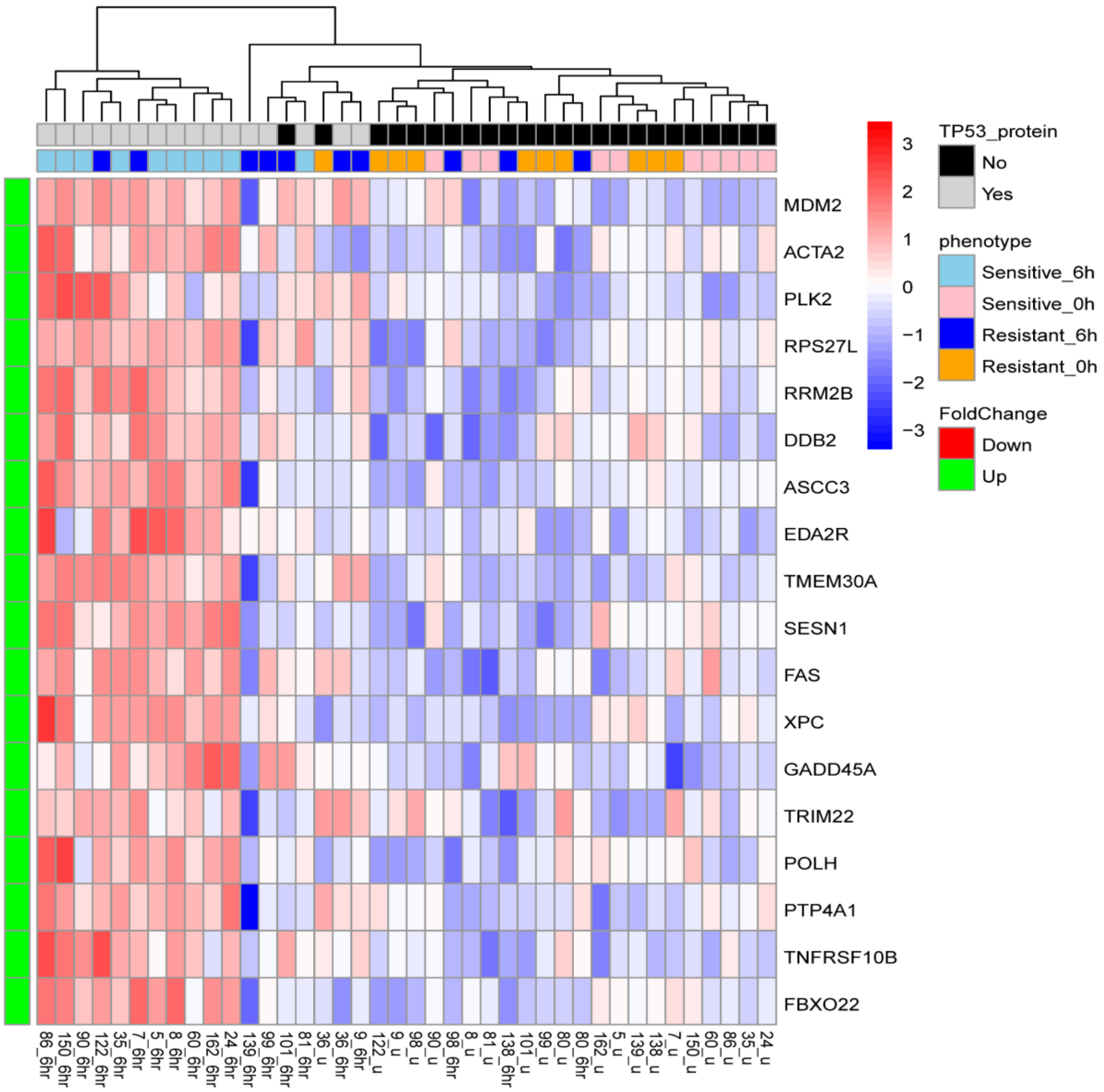

Supplementary Figure 3: Gene expression changes after 6h of MDM2 inhibitor treatment in primary human AML with WT *TP53* that are sensitive or resistant to MDM2 inhibitor induced apoptosis: Ten sensitive and 10 resistant primary human AML were purified and incubated with 10 µM of the MDM2 inhibitor MI219 for 0h, 2h and 6h. Gene expression was measured using GeneChip^{TM} Human Gene 1.0 ST Arrays (Affymetrix). In the scaled heatmap we display the genes with >1.5-fold changes at FDR < 0.2 at 6h in the sensitive cases compared with 0h (baseline) and the expression of these genes in the resistant cases at 0h and 6h, in which none showed significance at FDR < 0.2. The case ordering is based on unsupervised clustering indicated at the top. Phenotypes are indicated on the right. The expression of TP53 protein or lack thereof before and after MDM2 inhibitor treatment is indicated. Please also see Figure 3.

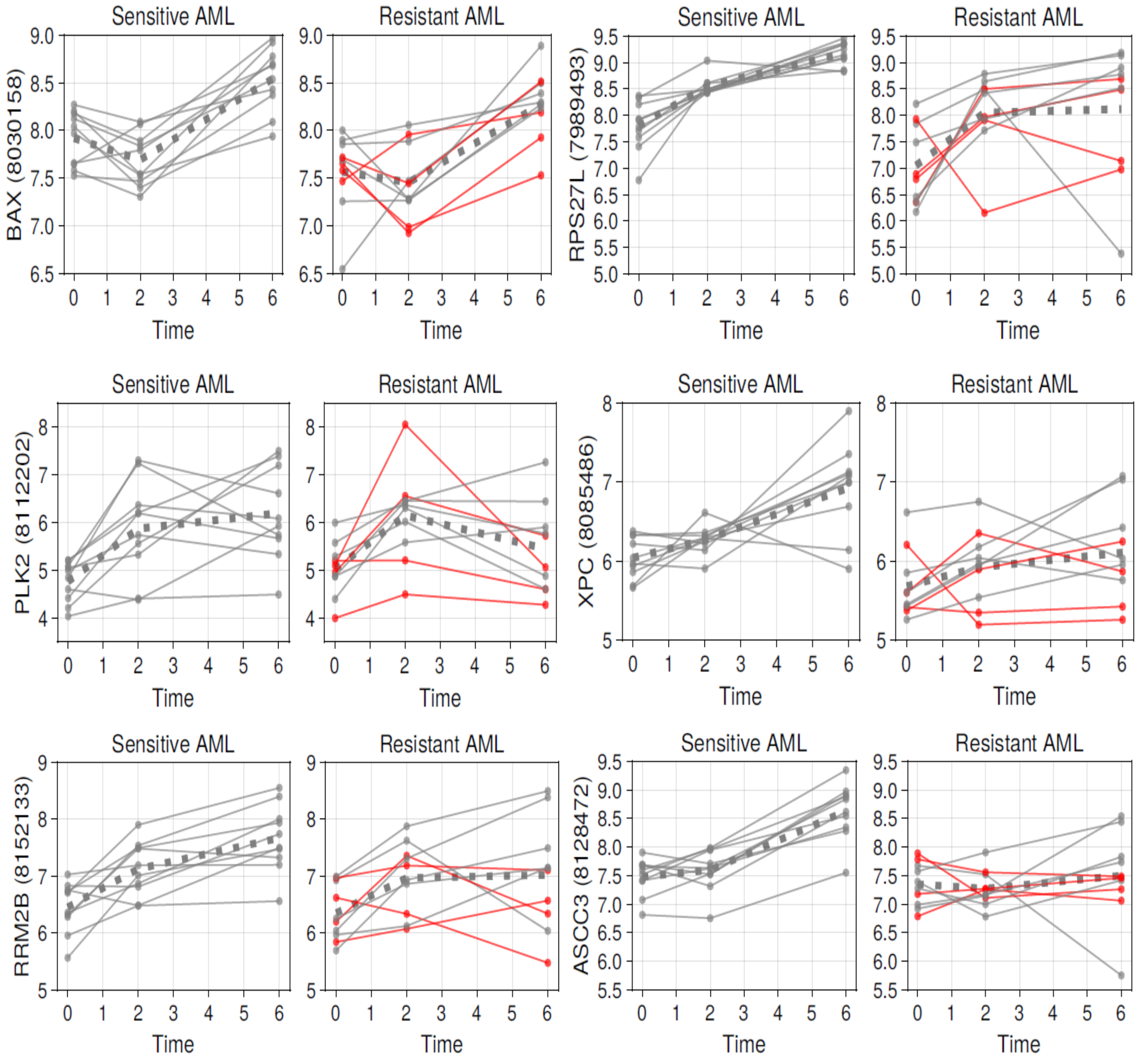

Supplementary Figure 4: Gene expression induction slopes in 10 sensitive and 10 resistant AML cases for known TP53 inducible genes: Ten sensitive and 10 resistant primary human AML all WT for *TP53* were purified and incubated with 10 µM of the MDM2 inhibitor MI219 for 0h, 2h and 6h. Gene expression was measured using GeneChip^{TM} Human Gene 1.0 ST Arrays (Affymetrix). Data for selected genes displayed in (Figure 3) are shown. Red lines: AML that lack TP53 protein expression at baseline and after MDM2 inhibitor treatment. X-axis: time in hours. Y-axis: Array hybridization values and probe ID. Dotted line represents the mean. Please also see Figure 4.

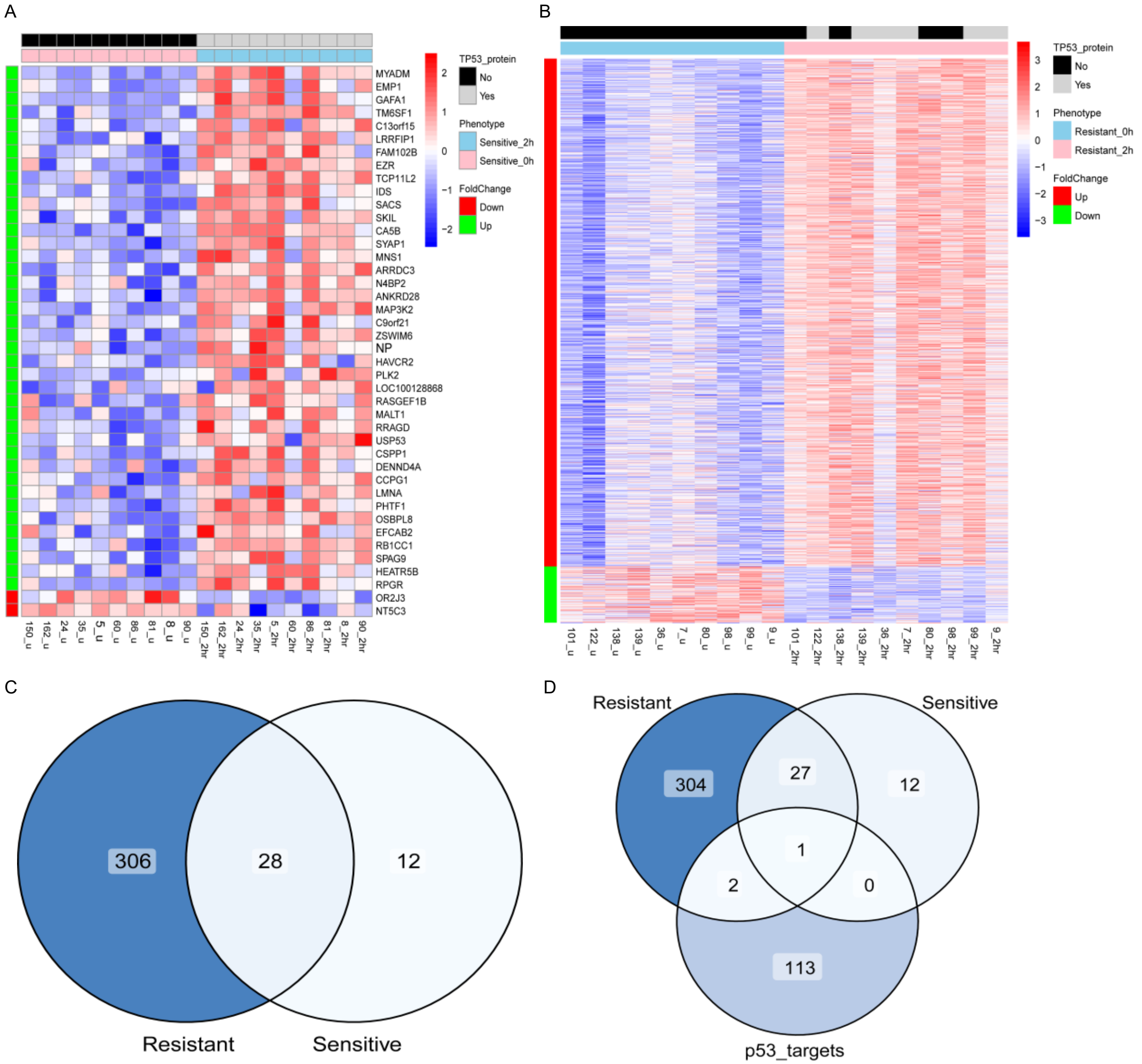

Supplementary Figure 5: <u>Gene expression changes after 2h of MDM2 inhibitor treatment in primary human AML with WT *TP53* that are sensitive or resistant to MDM2 inhibitor induced apoptosis:</u> Ten sensitive and 10 resistant primary human AML were purified and incubated with 10 μM of the MDM2 inhibitor MI219 for 0h, 2h and 6h. Gene expression was measured using GeneChip^{TM} Human Gene 1.0 ST Arrays (Affymetrix). In the scaled heatmap we display the genes with >2-fold changes at FDR < 0.1 at 2h compared with 0h (baseline) **(A)**: AML cases sensitive to MDM2 inhibitor induced apoptosis. **(B)**: AML cases resistant to MDM2 inhibitor induced apoptosis. **(C)**: Venn diagram of genes with >2-fold changes at FDR < 0.1 between sensitive and resistant cases. **(D)**: Venn diagram of genes with >2-fold changes at FDR < 0.1 between sensitive and resistant cases and a curated list of 116 high confidence *TP53* target genes as compiled by *Fischer Oncogene 2017*[1]. Phenotypes are indicated on the right. The expression of TP53 protein or lack thereof before and after MDM2 inhibitor treatment is indicated.

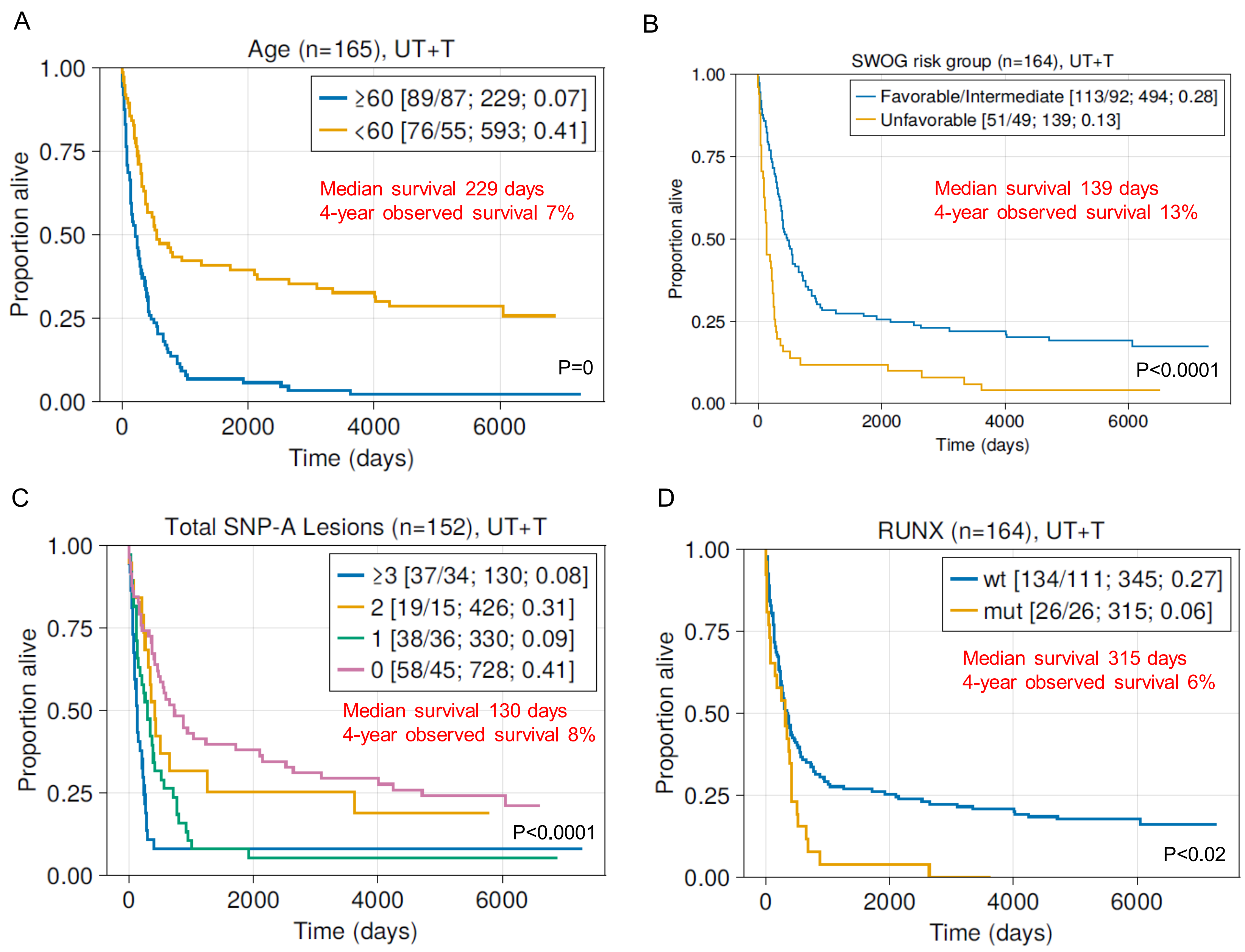

Supplementary Figure 6: <u>Survival of AML patients stratified by biomarkers (KM-plots):</u> A cohort of 165 AML patients with IC50 measurements was dichotomized using the indicated biomarkers. **(A)**: age, **(B)**: Southwest Oncology Group (SWOG) karyotypic risk group. **(C)**: SNP 6.0 SNP arrays based genomic lesions. **(D)**: *RUNX1* mutations. The median survival time in days and the surviving fraction of patients at 4 years is indicated. Survival time distributions were estimated using the Kaplan-Meier method, and comparisons between groups were made using log rank tests.
