# Supplementary material for: Patients with AML with WT TP53 but defective TP53-mediated apoptosis have a dismal survival
Source: JCI Insight. 2026 Jan 27;11(5):e197261. doi: 10.1172/jci.insight.197261 (PMC13041678; doi:10.1172/jci.insight.197261)

Full unedited gels for Figure 1: Sensitive AML

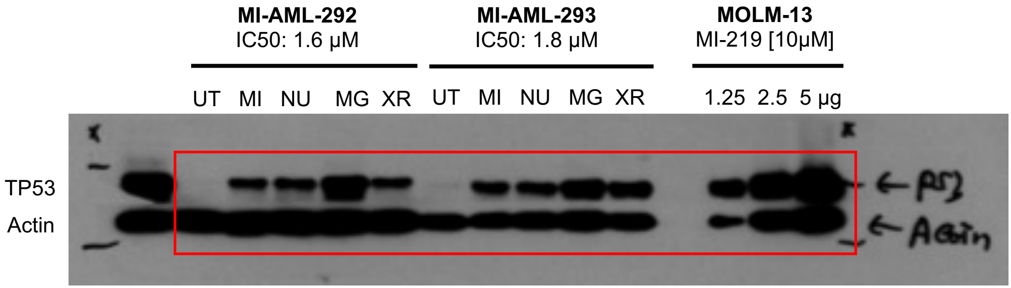

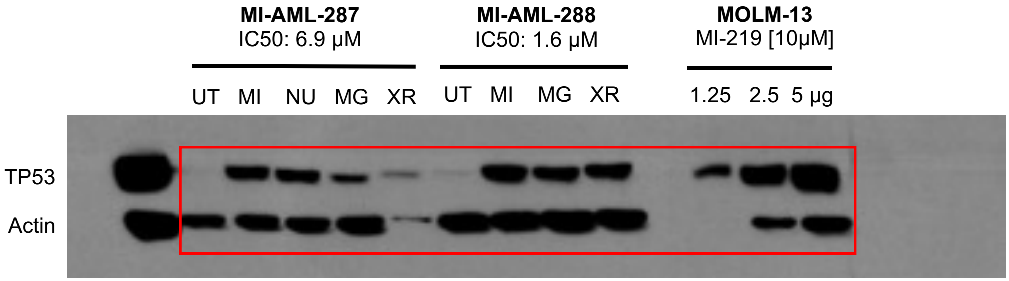

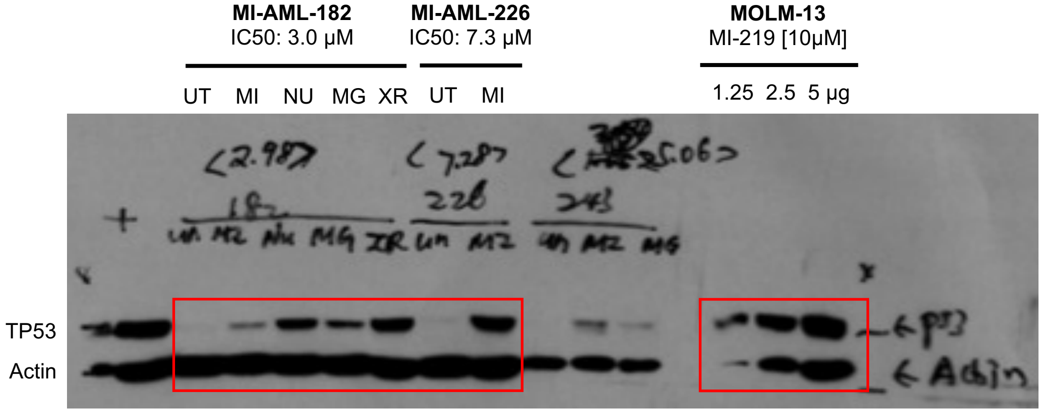

Full unedited gels for Figure 1: Sensitive AML (continued)

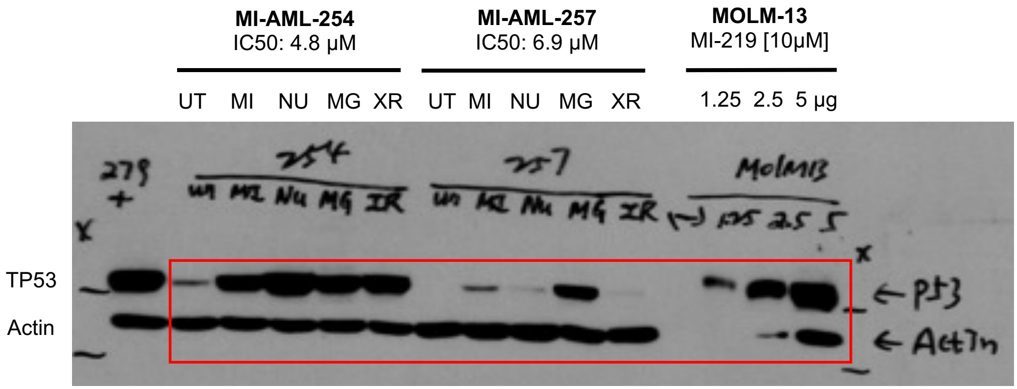

Full unedited gels for Figure 1: Resistant AML

MI-AML-263 IC50: > 20 µM | **MI-AML-267** IC50: 15.7 µM | **MOLM-13** MI-219 [10µM]

UT MI NU MG XR UT MI NU MG XR 1.25 2.5 5 µg

TP53

Actin

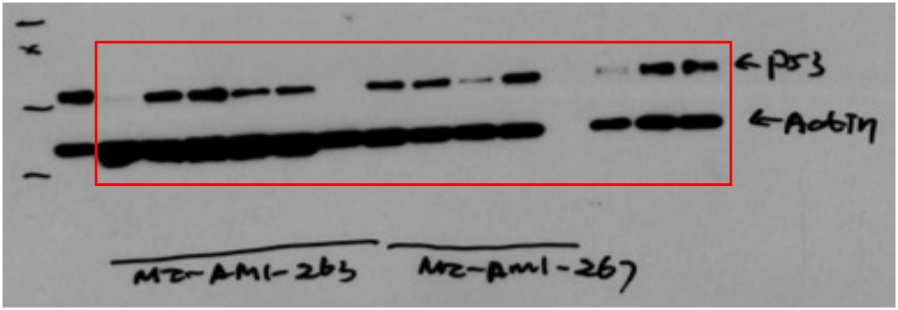

MI-AML-253 IC50: 11.1 µM | **MI-AML-255** IC50: > 20 µM | **MOLM-13** MI-219 [10µM]

UT MI NU MG XR UT MI NU MG XR 1.25 2.5 5 µg

TP53

Actin

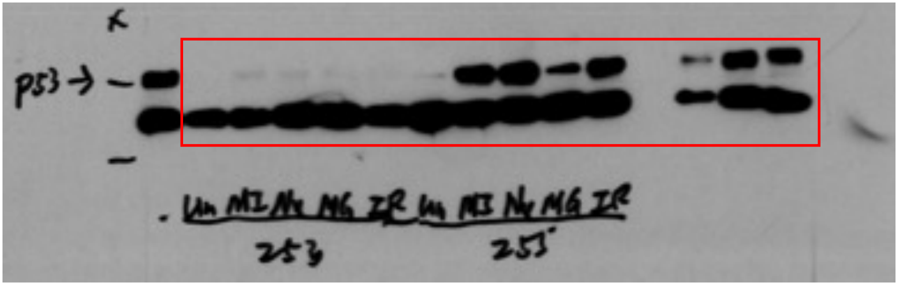

#68 10.9 µM | **#63** 20 µM | **#98** 20 µM | **#101** 20 µM | **#138** 20 µM | **MOLM-13** MI-219 [10µM]

UT MI UT MI UT MI UT MI UT MI 1.25 2.5 5 µg

TP53

Actin

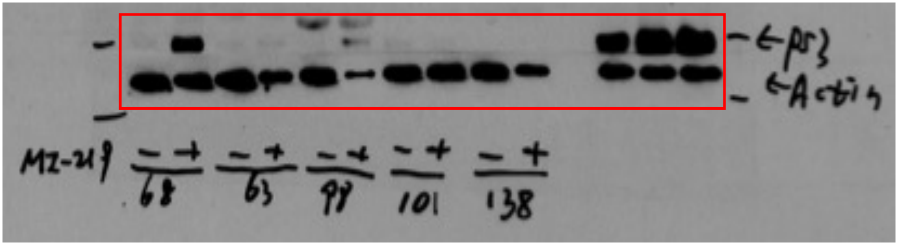

Full unedited gels for Figure 1: Resistant AML (continued)

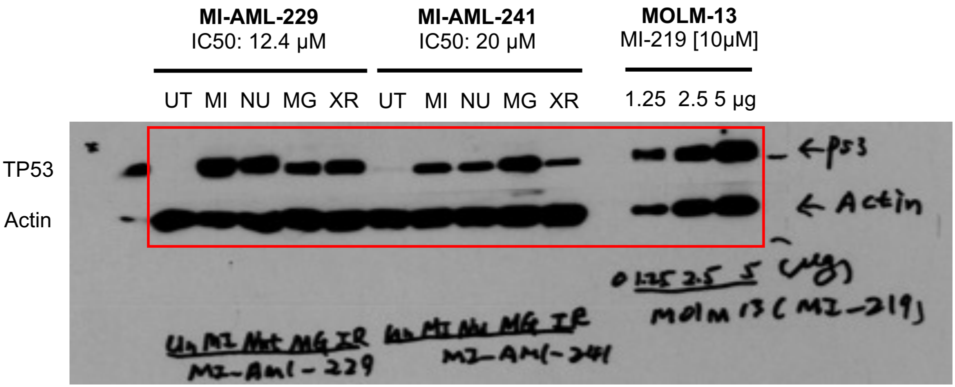

Full unedited gels for Supplementary Figure 1: Sensitive AML

MI-AML-251 IC50: 6.3 µM	MI-AML-252 IC50: 6.2 µM	MOLM-13 MI-219 [10µM]
UT MI NU MG XR	UT MI NU MG XR	1.25 2.5 5 µg

TP53
Actin

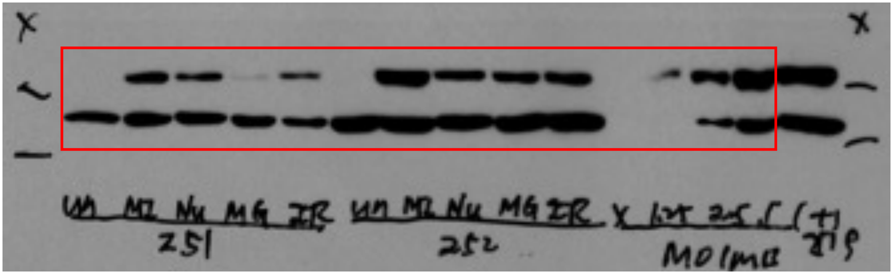

MI-AML-233 IC50: 8.9 µM	MI-AML-235 IC50: 2.1 µM	MOLM-13 MI-219 [10µM]
UT MI NU MG XR	UT MI NU MG XR	1.25 2.5 5 µg

TP53
Actin

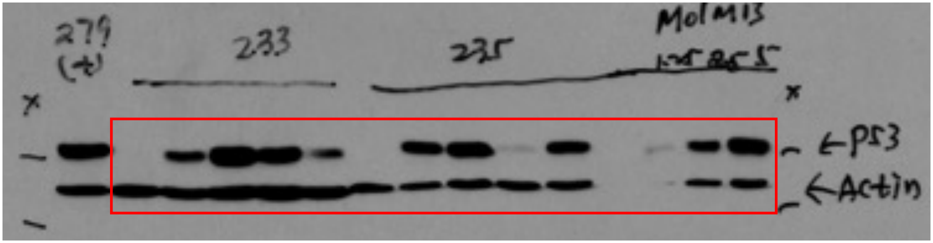

MI-AML-197 IC50: 2.8 µM	MI-AML-198 IC50: 6.7 µM	MOLM-13 MI-219 [10µM]
UT MI NU	UT MI NU	1.25 2.5 5 µg

TP53
Actin

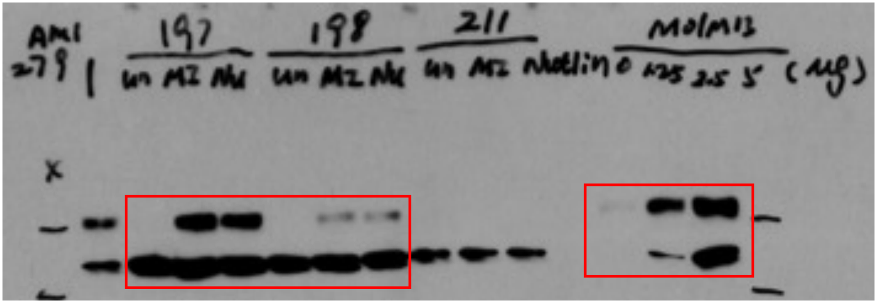

Full unedited gels for Supplementary Figure 1: Sensitive AML (continued)

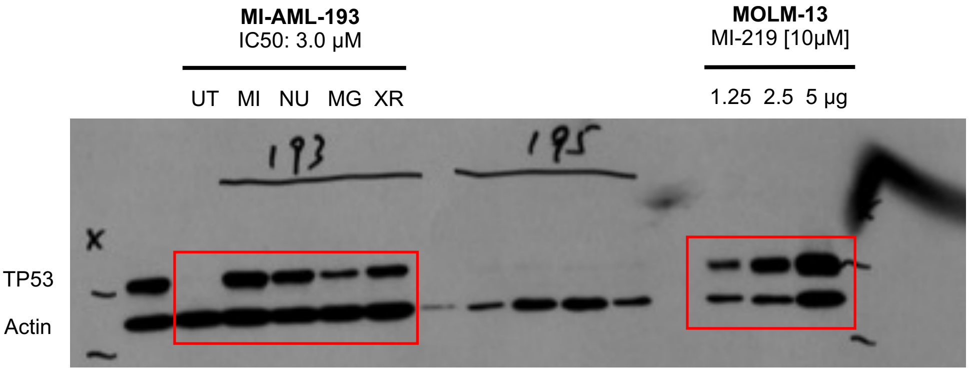

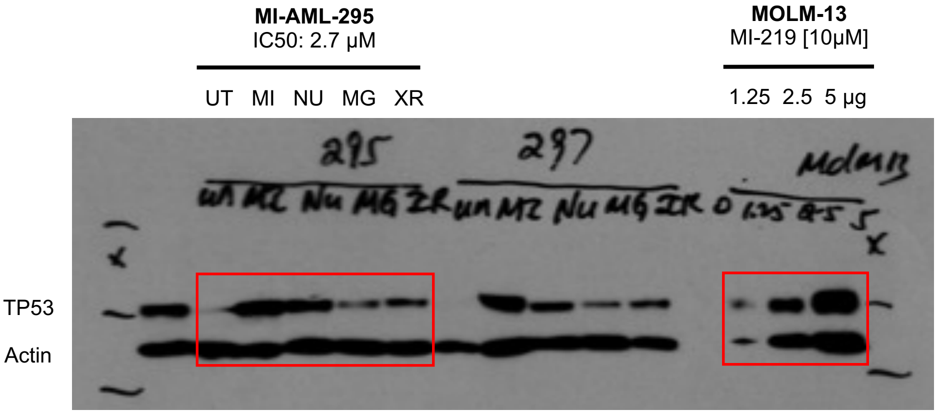

Full unedited gels for Supplementary Figure 1: Resistant AML

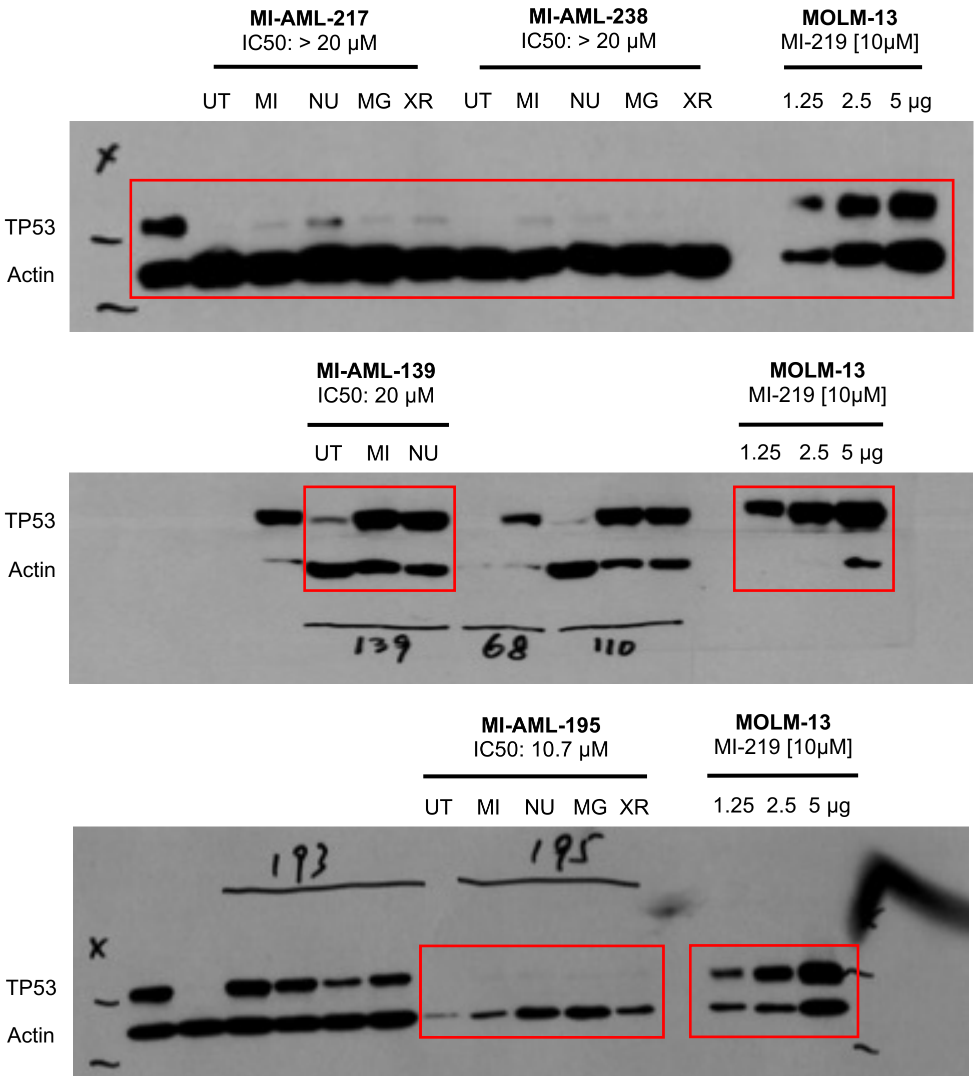

Full unedited gels for Supplementary Figure 1: Resistant AML (continued)

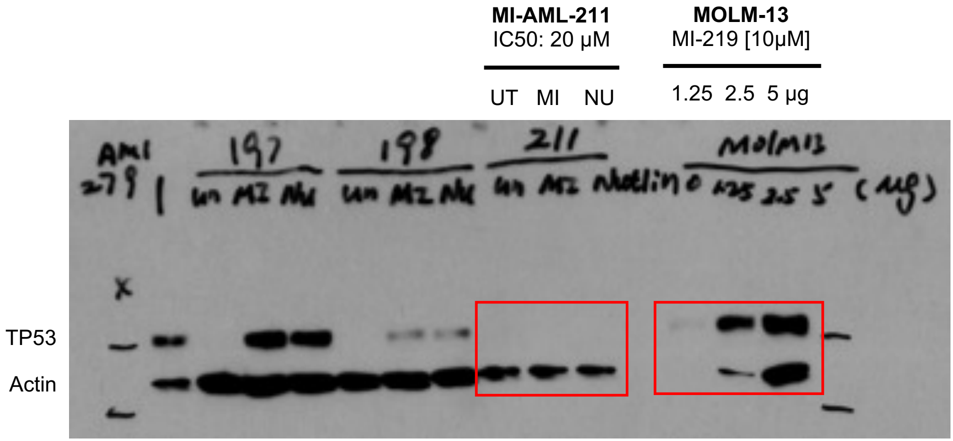

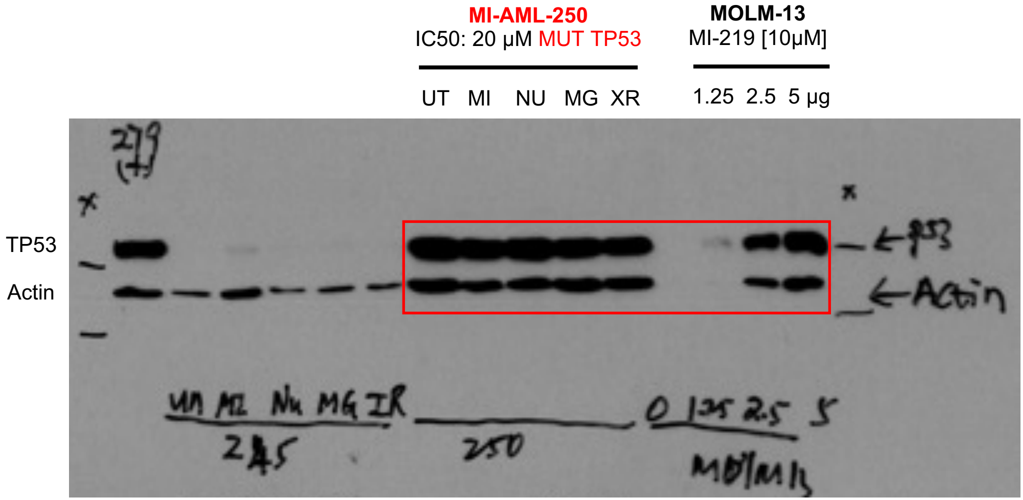

Supplement: Unedited blot and gel images [file jciinsight-11-197261-s024.pdf]
